# Correction: Rehman et al. The Multifaceted Role of Jasmonic Acid in Plant Stress Mitigation: An Overview. *Plants* 2023, *12*, 3982

**DOI:** 10.3390/plants15213412

**Published:** 2026-11-03

**Authors:** Muhammad Rehman, Muhammad Sulaman Saeed, Xingming Fan, Abdul Salam, Raheel Munir, Muhammad Umair Yasin, Ali Raza Khan, Sajid Muhammad, Bahar Ali, Imran Ali, Jamshaid Khan, Yinbo Gan

**Affiliations:** 1 Zhejiang Key Lab of Crop Germplasm, Department of Agronomy, College of Agriculture and Biotechnology, Zhejiang University, Hangzhou 310058, Chinamuhammadsulamansaeed2598@gmail.com (M.S.S.);; 2 Institute of Food Crops, Yunnan Academy of Agricultural Sciences, Kunming 650204, China; 3 Department of Botany, Kohat University Science and Technology, Kohat 26000, Pakistan; 4 Department of Biotechnology and Genetic Engineering, Kohat University Science and Technology, Kohat 26000, Pakistan

There were errors in the original publication [1]. Several references were inadvertently included during manuscript preparation and do not correspond to their associated text. The corrections are described below.

(1) In Section 7.1 (Drought Stress), reference 98 was inadvertently included and does not correspond to the associated text. The entry cited in error was “Vickers, N.J. Animal Communication: When I’m Calling You, Will You Answer Too? *Curr. Biol.* **2017**, *27*, R713–R715”. Reference 98 [2] has been replaced with the correct reference (given below reference 2), which directly reports jasmonic acid–induced inhibition of root growth and jasmonic acid–induced leaf senescence, including chlorophyll degradation. The in-text citation number and the associated sentence are unchanged and read:

“By applying a foliar spray of JA, the oxidative stress was alleviated and other harmful impacts were also increased because, due to the application of JA, the root growth was stunted and leaf and chlorophyll synthesis was also decreased in some plants [58,97,98].”

(2) In Section 7.6 (Mitigation of Heavy Metals Stress through JA), reference 124 was inadvertently included and does not correspond to the associated text. The entry cited in error was “Chandran, S.P.; Chaudhary, M.; Pasricha, R.; Ahmad, A.; Sastry, M. Synthesis of gold nanotriangles and silver nanoparticles using Aloevera plant extract. *Biotechnol. Prog.* **2006**, *22*, 577–583”. Reference 124 has been replaced with the original research articles [3,4], and the citation [24,34] in the same paragraph has been replaced with the original studies [5,6]. In addition, the gene name “GHAOS” has been corrected to “*GhAOC1*”, and the statement on JA levels in *TaAOS*-overexpressing tobacco has been corrected, as JA levels were not determined in that study. The corrected sentences read:

“The application of JA was also observed in tomato *SPR2* mutants which lacked jasmonic acid genes [124,125]. … Compared to JA signaling mutants, the transgenic plants have a highly overexpressing level of jasmonic acid biosynthetic genes with the increased level of JA and showed great resistance against heavy metal toxicity [126,127]. As an example, *Gossypium hirsutum GhAOC1* activated JA biosynthesis and showed improvement in the stress of the heavy metal. Moreover, the rate of sustainability of *GhAOC1* overexpression of transgenic plants under the treatment of copper conditions was two-fold more compared to the wild-type plants (Figure 7). … Furthermore, in tobacco plants the over-expression of *TaAOS* was investigated and enhanced zinc tolerance was observed compared to the control plants.”

(3) In Section 4 (Biosynthesis of JA), reference 53 has been replaced with reference 57. We also add 6 more references: reference 82 in the section 7, reference 4 in the section 7.1, reference 28 in the section 7.2, reference 10 in the section 7.3, reference 41 in the section 7.4 and reference 5 in the section 7.5.

The authors state that the scientific conclusions are unaffected. This correction was approved by the Academic Editor. The original publication has also been updated.
